# Colorectal cancer screening completion by patients due or overdue for screening after reminders: a retrospective study

**DOI:** 10.1186/s12885-023-10837-y

**Published:** 2023-05-01

**Authors:** Abdillahi M. Ahmed, Michael W. Bacchus, Stacy G. Beal, Katherine N. Huber, Ji-Hyun Lee, Jing Zhao, Thomas J. George, Maryam Sattari

**Affiliations:** 1 University of Florida College of Medicine, Gainesville, FL USA; 2 Department of Pathology, Immunology, and Laboratory Medicine, University of Florida College of Medicine, Gainesville, FL USA; 3 Division of General Internal Medicine, University of Florida College of Medicine, 1329 SW 16Th Street, Suite 5140, PO Box 103204, Gainesville, FL 32610 USA; 4 Department of Biostatistics, University of Florida, Gainesville, FL USA; 5 Division of Quantitative Sciences, University of Florida Health Cancer Center, Gainesville, FL USA; 6 Division of Hematology-Oncology, University of Florida College of Medicine, Gainesville, FL USA

**Keywords:** Colorectal cancer screening, Predictors, Clinician reminders, Patient reminders

## Abstract

**Background:**

Patient and clinician reminders were implemented as part of an adherence improvement project at University of Florida (UF) Internal Medicine Clinics. We sought to assess colorectal cancer (CRC) screening completion rates among patients not up-to-date with screening following distribution of reminders and to identify characteristics correlated with screening outcomes.

**Methods:**

Retrospective chart review was performed for patients not up-to-date with CRC screening for whom at least one reminder (patient and/or clinician) was issued in June 2018. The primary endpoint, the completion of a CRC screening test, is characterized as the ratio of completed screening tests to the number of patients not up-to-date with screening. All analyses were performed using R 4.0 software.

**Results:**

Of the 926 patients included, 403 (44%; 95% CI, 0.40–0.47) completed a CRC screening test within 24 months following a reminder. Family history of CRC (relative risk (RR) 1.33; *P* = 0.007), flu immunization within two years of the reminder (RR 1.23; *P* = 0.019), and receiving a patient reminder either alone (RR 1.62; *P* < 0.001) or in combination with a clinician reminder (RR 1.55; *P* = 0.006) were positively associated with CRC screening completion. Reporting being divorced, separated, or widowed was negatively associated with screening completion (RR 0.70; *P* = 0.004).

**Conclusion:**

Reminders, in particular patient reminders, seem to be an effective method to enhance screening among patients not up-to-date with CRC screening. This study suggests that reminder efforts should be focused at the level of the patients and provides insight on target populations for practical interventions to further increase CRC screening adherence.

## Background

Colorectal cancer (CRC) is the third most commonly diagnosed malignancy in the world and is associated with high morbidity and mortality [1]. CRC screening decreases both CRC incidence (via removal of pre-cancerous polyps) and mortality (by identifying the cancer at an earlier stage when treatment is more effective) [2]. Various medical societies, including the World Gastroenterology Organization, have issued CRC screening guidelines and recommendations in North America, Europe, and Asia [3]. The United States Preventive Services Task Force (USPSTF) strongly recommends that asymptomatic, average-risk individuals aged 50 to 75 undergo CRC screening, because of high certainty that the net benefit of screening is substantial in this group [4]. Recently, the USPSTF also added grade B recommendations to reduce the screening initiation age to 45 [4]. Grade B recommendations are issued when there is high certainty that the net benefit of a service is moderate or there is moderate certainty that the net benefit is moderate to substantial [4].

While CRC screening strategies (e.g. population-based versus opportunistic screening, modality of screening) as well as participation in screening vary geographically, enhancing screening participation is a global priority to further reduce CRC incidence and its associated morbidity and mortality [1]. In 2015, the National CRC Roundtable (NCCRT), a national coalition of public, private, and voluntary organizations, developed “80% by 2018”, an initiative with the aim of increasing CRC screening rates in the United States to 80% by 2018 [5]. The NCCRT recommended a multilevel approach to enhance CRC screening and prevention [5].

In 2015, the University of Florida (UF) College of Medicine in Gainesville, Florida joined the “80% by 2018” campaign, with the goal of enhancing CRC screening and prevention efforts. Various evidence-based multilevel interventions were developed and implemented as part of this initiative. Each initiative was evaluated and modified as needed. Two primary initiatives developed included patient and clinician reminders. Patient and clinician reminders have previously been associated with increased participation in CRC screening [6–10]. This study was conducted to assess CRC screening completion rates after clinician and/or patient reminders among patients not up-to-date with screening and to identify characteristics associated with screening completion in this non-adherent patient population.

## Methods

### Study design, setting, participants, and interventions

**Fig. 1:**
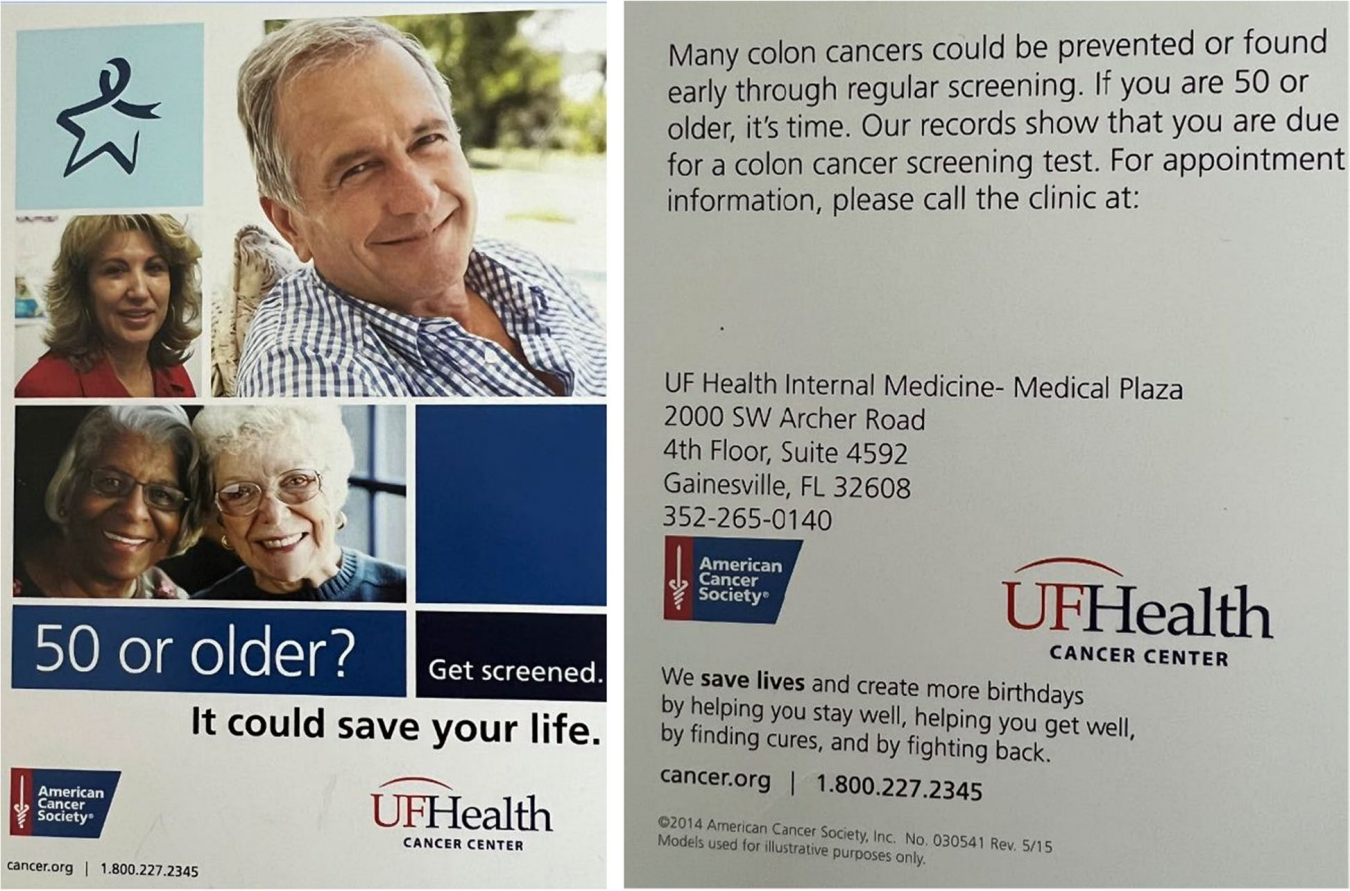
Reminder postcards mailed to patients not up to date with colorectal cancer screening

This was a non-randomized retrospective analysis of data collected following an observational practice improvement study in which reminders were provided based on sample sizes of convenience and patients’ CRC screening status. Patients eligible to receive patient reminders were prospectively identified on a quarterly basis by an algorithm that used the data within EPIC electronic medical records (EMR) to identify patients 50–75 years of age with upcoming appointments within the next 90 days at one of the Internal Medicine clinics at the University of Florida (UF) in Gainesville, Florida and not otherwise up-to-date with CRC screening. These individuals were mailed a patient postcard generated in partnership with the Florida Department of Health and the American Cancer Society (Fig. 1). Patients eligible for clinician reminders were identified on a daily basis by a trained clinical navigator who manually reviewed the charts of all patients 50–75 years of age participating in a UF Internal Medicine clinic visit. The clinical navigator determined CRC screening status of each patient and sent reminders to their clinicians for patients not up-to-date with CRC screening. The clinician reminders were typically sent one business day prior to the day of the clinic visit for each patient, independent of whether the patient previously had or had not received a patient reminder. The goal was to generate both a clinician and patient reminder for every patient with a clinic visit who was due or overdue for CRC screening. However, due to the fluidity of clinic schedules (e.g. clinic cancelations, urgent visits), some patients only had one type of reminder generated.

The 2016 USPSTF guidelines and results of the most recent available screening tests were used to determine each patient’s up-to-date status with screening (i.e., a normal guaiac-based fecal occult blood test [gFOBT] within 12 months, a fecal immunochemical test [FIT] within 12 months, a Cologuard [stool FIT-DNA test] within 36 months, a normal colonoscopy within 10 years, a computed tomography [CT] colonography within 5 years, or a flexible sigmoidoscopy within 5 years) [4]. Data collection was conducted via review of EMR for each patient who was mailed a patient reminder postcard in June 2018 or whose clinician received a clinician reminder within EMR in June 2018. Patients who were subsequently determined to be up-to-date on screening at the time of the reminder (*n* = 134), deceased at the time of data collection (*n* = 43), or with a primary care clinician outside of the UF Health network at the time of data collection (*n* = 13) were excluded from the study.

The charts of the patients who met eligibility criteria were reviewed to determine if they had completed a CRC screening test within 24 months after the reminder (i.e. between June 2018 and June 2020). Each patient was categorized as having received one of three reminder types: (1) patient reminder only defined as a reminder postcard, (2) clinician reminder only defined as an EMR reminder sent to the clinician, and (3) both clinician and patient reminders. The patients who received the same type of reminder more than once in the study period were counted only once in that reminder category. Additional information collected via review of the EMR included demographic variables (e.g., age, sex, race, ethnicity, state of residence, zip code, highest level of education completed, tobacco use, and alcohol use), past medical history (heart disease, cancer, colorectal disease, mood disorders, and flu vaccination within the past two years), and documented family history of CRC. This study was approved by the UF Institutional Review Board and granted an exemption from requiring informed consent. The study was performed in accordance with the Declaration of Helsinki.

### Study end points

The primary outcome was completion of CRC screening within 24 months following receipt of reminder(s). Secondary end-points were variables associated with CRC screening completion.

### Statistical analysis

Descriptive statistics are reported as mean and standard deviation (SD) for continuous variables and frequencies and proportions for categorical variables. The primary endpoint, the completion of CRC screening test, is characterized as the ratio of completed screening tests to the number of patients not up-to-date with screening. The screening rate and its 95% confidence interval (CI) was calculated based on the exact binomial distribution. Relative risk (RR) of potential variables for completion of CRC screening was calculated using both univariable and multivariable analysis in generalized linear model (GLM) with binomial log link function. Backward selection was used to select variables for the multivariable model. The estimated RR and its 95% CI were reported from both models. All analyses were performed using R 4.0 software (R core team, 2020) [11].

## Results

**Fig. 2:**
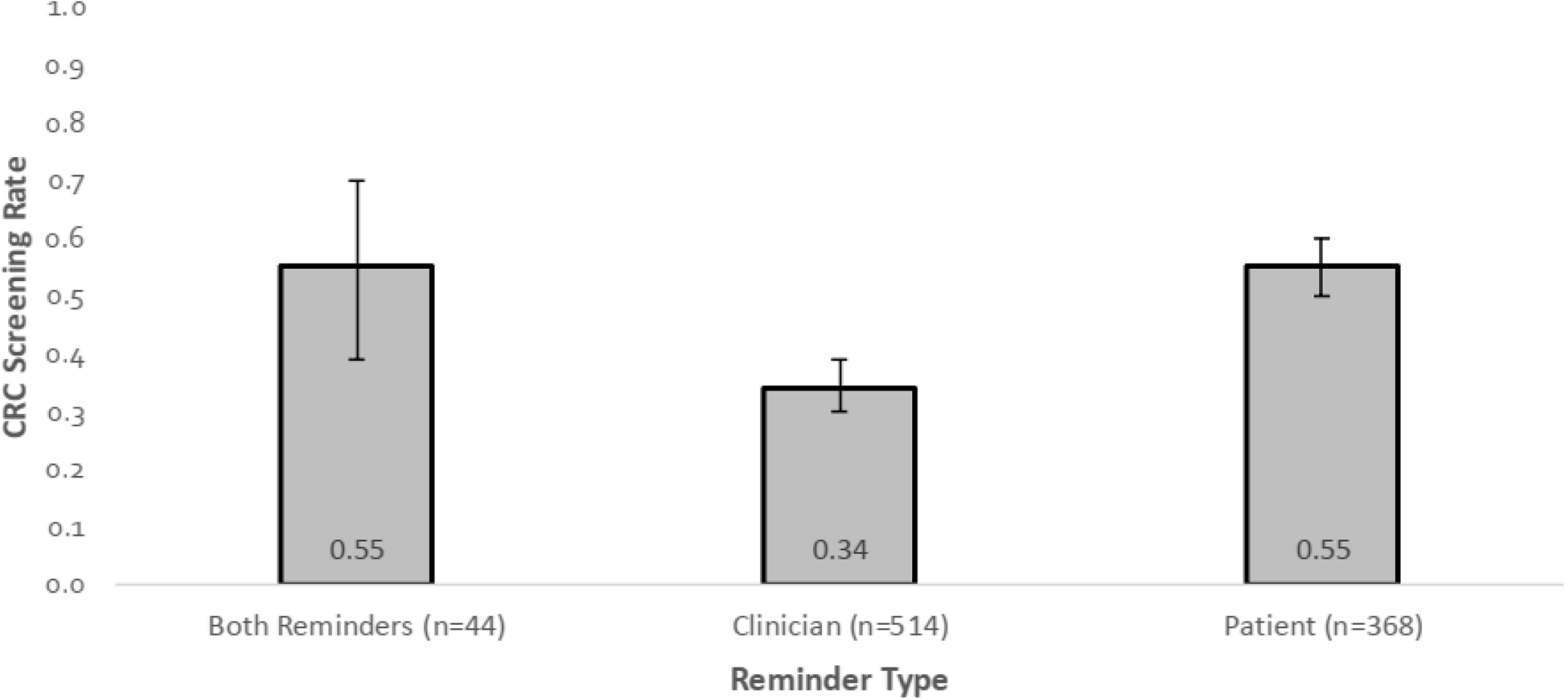
Colorectal cancer screening completion among each reminder type group. A CRC screening test was completed by 24 patients in the group with both reminder types, 177 in the clinician reminder only group, and 202 in the patient reminder only group

The study included 926 patients, 368 (40%) in the patient reminder only group, 514 (55%) in the clinician reminder only group, and 44 (5%) in the group with both types of reminders. The mean age of the participants was 62.7 years and the majority were Caucasian (80.8%), female (61%), and married (63.4%) (Table 1). A CRC screening test was subsequently completed by 403 (44%; 95% CI, 0.40–0.47) of these patients, 202 (55%; 95% CI, 0.50–0.60) in the patient reminder only group, 177 (34%; 95% CI, 0.30–0.39) in the clinician reminder only group, and 24 patients (55%; 95% CI, 0.39–0.70) in the group with both reminder types (Fig. 2).

**Table 1:** Baseline patient characteristics (*n* = 926)

**Characteristic**	
Age mean (SD)	62.7 (7.5)
Female n (%)	564 (61.0)
Race n (%)^a^	
Black or African American	122 (15)
White	658 (80.8)
Others	34 (4.2)
Missing	112
Hispanic or Latino n (%)	38 (4.4)
State of residence n (%)	
Florida	909 (99)
Zip n (%)^b^	
Metropolitan area	776 (84.6)
Micropolitan	76 (8.3)
Small town / Rural area	65 (7.1)
Insurance n (%)	
Medicaid	25 (2.8)
Medicare	386 (42.7)
Medicare/Medicaid	37 (4.1)
No insurance	12 (1.3)
Private	444 (49.1)
Marital Status n (%)^c^	
Married or with Significant other	555 (63.4)
Single	182 (20.8)
Other (separated, divorced, or widowed)	138 (15.8)
Education n (%)	
High school or equivalent	3 (14.3)
Some college	4 (19)
Bachelor’s degree	1 (4.8)
Graduate or professional degree	13 (61.9)
Missing	905
Clinician level n (%)	
Faculty physician	727 (82.2)
Physician assistant	1 (0.1)
Resident physician	156 (17.6)
Tobacco use n (%)	
Current	86 (9.3)
Former	310 (33.5)
Never	528 (57.1)
Alcohol use n (%)	
Current	435 (48.2)
Former	449 (49.7)
Never	19 (2.1)
History of heart disease n (%)	279 (30.2)
History of cancer n (%)	
Breast cancer	42 (4.5)
Lung cancer	8 (0.9)
Prostate cancer	14 (1.5)
Other cancer	157 (17)
None	704 (76.1)
History of Colorectal Disease n (%)	372 (40.2)
Documented Family history of CRC n (%)	116 (13.7)
Received a flu shot within two years n (%)	687 (75.1)
History of mood disorder n (%)	342 (37.1)

*CRC* Colorectal cancer, *SD* Standard deviation

^a^Three racial categories were created: (1) White, (2) Black or African American, and (3) Other (Asian, American Indian, Alaska Native, Native Hawaiian, other Pacific Islander)

^b^Numerical zip codes were converted to categories of Metropolitan, Micropolitan, and Small town/Rural based on https://depts.washington.edu/uwruca/ruca-codes.php [12]

^c^Three marital status groups were created: (1) Single, (2) Married or with a significant other, and (3) Other (divorced, separated, or widowed)

Univariate results are summarized in Table 2. Multivariable analysis demonstrated a statistically significant association between CRC screening completion and four variables: family history of CRC, influenza vaccination, reminder type, and marital status (Table 3). Patients with a documented family history of CRC were more likely to have completed a CRC screening test (RR 1.33; 95% CI, 1.09–1.65; *P* = 0.007), compared to patients without a documented family history of CRC. Influenza vaccination within the past two years was also associated with increased likelihood of screening completion (RR 1.23; 95% CI, 1.03–1.44; *P* = 0.019). Patients who received a patient reminder only (RR 1.62; 95% CI, 1.38–1.90; *P* < 0.001) as well as the group with both reminder types (RR 1.55; 95% CI 1.09–2.03; *P* = 0.006) were more likely to complete screening, compared to the clinician reminder only group.

**Table 2:** Univariate analysis for CRC completion

Variable	RR (95% CI)	*P* value
Age	1 (0.99,1.01)	0.720
Gender – male, compared to female	0.94 (0.8, 1.09)	0.399
Race		
Black or African American	Reference	
White	1.05 (0.85, 1.34)	0.669
Other	0.99 (0.59, 1.49)	0.948
Ethnicity—Not Hispanic or Latino Ethnicity	1.05 (0.75, 1.62)	0.811
Tobacco use		
Current	Reference	
Former	1.3 (0.95, 1.86)	0.122
Never	1.41 (1.06, 2)	0.033
Alcohol use		
Current	Reference	
Former	0.88 (0.76, 1.02)	0.088
Never	0.56 (0.22, 1.03)	0.129
Marital Status		
Married or with a significant other	Reference	
Single	0.92 (0.75, 1.11)	0.400
Other (divorced, separated, or widowed)	0.77 (0.6, 0.97)	0.037
Medical insurance		
Medicare/Medicaid	Reference	
Private	1.07 (0.92, 1.24)	0.390
None	0.4 (0.07, 1.03)	0.151
Urbanicity		
Metropolitan	Reference	
Micropolitan	0.79 (0.56, 1.06)	0.149
Small town or rural area	0.93 (0.67, 1.22)	0.629
History of heart disease	0.95 (0.8, 1.11)	0.514
Cancer history		
No cancer history	Reference	
History of breast cancer	0.87 (0.55, 1.23)	0.492
History of lung cancer	1.71 (0.93, 2.21)	0.01
History of prostate cancer	1.31 (0.72, 1.86)	0.257
History of other cancer	0.95 (0.76, 1.15)	0.596
History of colorectal disease	0.82 (0.7, 0.95)	0.011
History of mood disorder	0.94 (0.8, 1.1)	0.449
Family history of CRC	1.36 (1.13, 1.61)	0.001
Flu vaccination within 2 years	1.35 (1.12, 1.66)	0.003
Clinician type – resident, compared to faculty	0.99 (0.8, 1.19)	0.906

*CI* Confidence interval, *CRC* Colorectal cancer, *RR* Relative ratio

**Table 3:** Multivariable analysis for colorectal cancer completion

Variable	Relative Risk (95% CI)	*P* value
Reminder Groups		
Both reminder types	1.55 (1.09, 2.03)	0.006
Patient reminder only	1.62 (1.38, 1.90)	< 0.001
Clinician reminder only	Reference	
Family history		
Documented family history of CRC	1.33 (1.09, 1.65)	0.007
No documented family history of CRC	Reference	
Influenza Vaccination		
Received influenza immunization within 2 years	1.23 (1.03, 1.44)	0.019
Did not receive influenza immunization within 2 years	Reference	
Marital Status		
Single	0.86 (0.70, 1.02)	0.108
Other (divorced, separated, or widowed)	0.70 (0.54, 0.88)	0.004
Married or with a significant other	Reference	

*CI* Confidence interval, *CRC* Colorectal cancer

While there was no statistically significant difference between screening completion rates for single and married individuals, patients in the “Other” marital group (divorced, separated, or widowed) were less likely to complete screening (RR 0.70; 95% CI, 0.54–0.88; *P* = 0.004), compared to the group that reported being married or with a significant other. The following variables did not have a statistically significant association with CRC screening completion: age, gender, race, ethnicity, smoking, medical insurance, urbanicity, clinician type (resident physician vs. faculty physician), and personal history of colorectal disease, heart disease, cancer, or mood disorder. Education level was not included in the analysis due to multiple missing values in the EMR.

## Discussion

Overall, 44% of patients who were due or overdue for CRC screening became adherent in this study by completing a screening test. It is important to note that this study only targeted patients who were not up-to-date with CRC screening at baseline, thus we were able to narrow the gap in the overall screening participation and improve our goals of bringing more patients into adherence with CRC screening. However, the remaining 56% of patients included in this analysis continued to remain out of date with screening, despite the multi-layered interventions employed in our clinics to enhance CRC screening and prevention. Developing more effective strategies to engage these remaining patients is an essential step in further enhancing CRC screening, and thereby reducing the incidence, morbidity, disparities, and mortality of CRC. Other strategies suggested for increasing screening uptake include patient navigation and other interventions for structural barriers and community resource gaps, raising awareness of CRC screening [by individual education (e.g., health counseling and clinicians), recommendations by modalities such as letters and phone calls, and the use of small media (e.g., brochures and newsletters)], population-based outreach screening (e.g. mailing a FIT kit to eligible individuals), clinical decision support, providing a choice of screening tests, patient- and clinician-based behavioral interventions, tracking screening metrics, and ensuring access to colonoscopy irrespective of socioeconomic status [5–10, 13].

This study supports and adds to existing literature regarding factors related to patient participation in CRC screening. Consistent with previous studies among all screen-eligible patients [14], patients in this study with a documented family history of CRC were 33% more likely to complete CRC screening following any form of reminder. These individuals might have higher perceived susceptibility to CRC and be more aware of CRC, the impact it has when diagnosed as well as the need for and benefits of screening and early detection. Similar to studies among screen-eligible patients, study participants who had received an influenza vaccination within the past two years were 23% more likely to complete a CRC screening test following any form of a reminder [14]. This finding might reflect a correlation between concern about cancer screening and utilization of other preventive health services. Individuals who were divorced, separated, or widowed were 30% less likely to complete screening, compared to individuals who were married or had a significant other. These findings are consistent with previous research in the general population that has demonstrated increased odds of cancer screening among married and unmarried couples, compared to divorced, widowed, and separated participants [15]. While the underlying mechanisms of the association between marital status and health-seeking behaviors is not well understood, possible explanations include social control, where one partner attempts to control the individual’s behaviors to keep him or her healthy, relationship-centered interests of couples instead of person-centered interests, and partner’s influence on health-seeking behaviors (e.g. setting up an appointment with a primary care physician) [15, 16]. Recognition of these variables might benefit subgroups who underutilize screening. For example, patients without family history of CRC, those who are not up-to-date on their influenza vaccination, and those who are separated, divorced, or widowed might specially benefit from targeted interventions to raise awareness of CRC and benefits of appropriate cancer screening. Collectively, the identification of these variables might lead to the identification of common barriers that preclude or contribute to non-adherence with other medical recommendations.

While primary care provider recommendations have been found to be one of the most effective tools for increasing CRC screening [17, 18] and physicians have reported reminder systems as a facilitator for screening [19, 20], the clinician reminder only group in this study demonstrated the lowest screening completion rate (34%). Physician-related barriers to screening recommendation include lack of time during clinic visits and forgetfulness [19]. Due to the retrospective nature of this study, it is unclear whether these same barriers resulted in lack of clinician-patient discussion about the due/overdue screening status and CRC screening recommendations. Moreover, the clinician reminders that were used were not “active” reminders and did not require physicians to respond. Therefore, busy clinicians could have missed or disregarded these reminders. Prior research also suggests that physicians prefer colonoscopy for CRC screening, while patients might prefer less invasive and/or more convenient screening modalities [6, 7, 10]. One study demonstrated increased number of colonoscopy orders after provider reminders without a statistically significant increase in completed colonoscopies [10]. Thus, intent to screen does not always translate to actual screening completion. The number and types of screening tests ordered were not assessed in this study, but the current findings may also reflect the need for improvement in clinician-patient communication regarding CRC and available modalities for screening.

We observed higher screening completion rates (55%) among the patient reminder only group. These results are consistent with the study by Sequist et al. that demonstrated higher CRC screening completion rates (43.7%) after patient reminders among patients overdue for screening, compared to physician reminders (39.6%) [10]. Patient-related barriers to CRC screening include lack of motivation, absence of awareness of the need for screening, and lack of receiving health care provider screening recommendations [21]. Patient requests are one of the physician-related facilitators for CRC screening [19, 20]. The postcard reminders mailed to patients in this study might have raised their awareness of CRC and need for screening and increased their motivation to discuss screening with their clinician and request a screening test. While there were only 44 patients in the group with both reminders, we observed similar rates of screening completion among the patient reminder only group and the group with both types of reminders. Sequist et al. also found similar CRC screening completion rates after patient reminders only (43.7%) and after both patient and physician interventions (44.2%) [10]. These findings suggest that in settings with limited resources, prioritizing patient-based interventions is a reasonable consideration.

### Limitations

One limitation of this study is the unavailability or inconsistent availability of important patient variables in the EMR, such as income and educational level. Also, we did not collect data regarding prior CRC screening behavior of the study participants. Therefore, this study was not able to assess the association of these variables, which might be confounders, with CRC screening participation. Similarly, race and ethnicity were not consistently documented in the EMR. The generalizability of our findings is also limited by the exclusivity of this research to the UF Internal Medicine clinics. Even within UF, we might have inadvertently missed patients who did not have scheduled appointments because they were healthy and/or uninsured/underinsured as most of the patients included in this study had some type of medical insurance. Another potential limitation is the small size of the group with both patient and clinician reminders. While our patient reminder postcard intervention could be implemented across a wide range of health care settings, generating a list of patients not up-to-date with screening requires reliable data and the information technology resources to create the list which may not be available in all centers. Lastly, the retrospective nature of the analysis precludes the ability to derive true causality between the identified factors and the screening completion outcome, absent other uncontrolled variables that could impact the results. Future prospective studies that randomize reminder types would provide further insight into the relative efficacy of different reminder types. However, we believe the data represent a low-cost, systematic intervention that at best, may have been associated with a near halving of the non-adherence in CRC screening rates and engages patients directly in their healthcare decision making.

## Conclusion

Increasing CRC screening rates is an essential step in reducing the incidence, morbidity, disparities, and mortality of CRC. This study demonstrates effectiveness of reminders to patients and/or clinicians in increasing screening rates among patients otherwise due or overdue for CRC screening. Furthermore, while this was not a randomized control study, patient reminders (with or without clinician reminders) were associated with higher CRC screening completion rates, suggesting the importance of focusing reminders at the level of patients. Our findings also provide insight on target populations for practical interventions to further increase CRC screening adherence.

## Data Availability

The datasets used and analysed during the current study are available from the corresponding author on reasonable request.

## Abbreviations

CI: Confidence interval
CRC: Colorectal cancer
CT: Computed tomography
EMR: Electronic medical records
FIT: Fecal immunochemical test
gFOBT: Guaiac-based fecal occult blood test
GLM: Generalized linear model
NCCRT: National CRC Roundtable
RR: Relative risk
SD: Standard deviation
UF: University of Florida
USPSTF: The United States Preventive Services Task Force
